# Fibular dimelia and mirror polydactyly of the foot in a girl presenting additional features of the VACTERL association

**DOI:** 10.1590/S1516-31802010000200011

**Published:** 2010-03-04

**Authors:** Pricila Bernardi, Carla Graziadio, Rafael Fabiano Machado Rosa, Juliana Nunes Pfeil, Paulo Ricardo Gazzola Zen, Giorgio Adriano Paskulin

**Affiliations:** I MD. Clinical geneticist, Universidade Federal de Ciências da Saúde de Porto Alegre (UFCSPA) and Complexo Hospitalar Santa Casa de Porto Alegre (CHSCPA), Porto Alegre, Rio Grande do Sul, Brazil.; II MD. Assistant professor and Clinical Geneticist, Universidade Federal de Ciências da Saúde de Porto Alegre (UFCSPA) and Complexo Hospitalar Santa Casa de Porto Alegre (CHSCPA), Porto Alegre, Rio Grande do Sul, Brazil.; III MD. Postgraduate student and clinical geneticist, Universidade Federal de Ciências da Saúde de Porto Alegre (UFCSPA) and Complexo Hospitalar Santa Casa de Porto Alegre (CHSCPA), Porto Alegre, Rio Grande do Sul, Brazil.; IV Medical student, Universidade Federal de Ciências da Saúde de Porto Alegre (UFCSPA), Porto Alegre, Rio Grande do Sul, Brazil.; V PhD. Adjunct professor of Clinical Genetics and professor of the Postgraduate Program on Pathology and Clinical Genetics, Universidade Federal de Ciências da Saúde de Porto Alegre (UFCSPA) and Complexo Hospitalar Santa Casa de Porto Alegre (CHSCPA), Porto Alegre, Rio Grande do Sul, Brazil.; VI PhD. Associate professor of Clinical Genetics and professor of the Postgraduate Program on Pathology, Clinical Genetics and Cytogenetics, Universidade Federal de Ciências da Saúde de Porto Alegre (UFCSPA) and Complexo Hospitalar Santa Casa de Porto Alegre (CHSCPA), Porto Alegre, Rio Grande do Sul, Brazil.

**Keywords:** Polydactyly, Tibia, Lower extremity deformities, congenital, Upper extremity deformities, congenital, Misoprostol, Polidactilia, Tíbia, Deformidades congênitas das extremidades inferiores, Deformidades congênitas das extremidades superiores, Misoprostol

## Abstract

**CONTEXT:**

The association between fibular dimelia and mirror polydactyly of the foot is considered to be a very rare lower-limb abnormality. On the other hand, VACTERL is an acronym for a nonrandom association of congenital anomalies for which the etiology is still poorly understood.

**CASE REPORT:**

The patient was a seven-month-old white girl whose mother had used misoprostol in the second month of pregnancy to induce abortion. On clinical evaluation, she was small for her age and presented hypotonia, anteverted nares, long philtrum and carp-like mouth. Her left hand had a reduction defect, with absence of the extremities of the second, third and fifth fingers and camptodactyly of the fourth finger. The ipsilateral lower limb presented significant shortening, especially rhizomelic shortening. Her left foot had a mirror configuration with seven toes and no identifiable hallux. The pelvis was hypoplastic. Esophageal atresia with tracheoesophageal fistula and imperforate anus were detected during the neonatal period. Abdominal ultrasound identified agenesis of the right kidney and left pyelocaliceal duplication. Radiographic evaluation on the left side showed iliac and femoral hypoplasia, absence of the tibia with a duplicated fibula and seven metatarsals and toes with no identifiable hallux on the foot. Echocardiography demonstrated an atrial septal defect. Based on the literature, we believe that the spectrum of malformations presented by our patient may be related to the vascular disruptive effect of the misoprostol. However, we cannot rule out the possibility that this association might simply be a coincidence.

## RESUMO

**CONTEXTO:**

A associação entre dimelia fibular e polidactilia em espelho do pé é considerada uma anormalidade de membro inferior bastante rara. Por outro lado, VACTERL é um acrônimo para uma associação não aleatória de anomalias congênitas cuja etiologia ainda é pouco compreendida.

**RELATO DO CASO:**

A paciente era uma menina branca de sete meses de idade, cuja mãe utilizou misoprostol no segundo mês de gravidez para indução de aborto. Na avaliação clínica, ela era pequena para a idade e apresentava hipotonia, narinas antevertidas, filtro longo e boca em carpa. A mão esquerda apresentava um defeito de redução com ausência das extremidades do segundo, terceiro e quinto dedos e camptodactilia do quarto. O membro inferior ipsilateral apresentava um importante encurtamento, especialmente rizomélico. O pé possuía uma configuração em espelho com sete dedos e nenhum hálux identificável. A pelve era hipoplásica. Atresia de esôfago com fístula traqueoesofágica e imperfuração anal foram detectadas durante o período neonatal. O ultrassom abdominal identificou agenesia do rim direito e duplicidade pielocalicial à esquerda. A avaliação radiográfica mostrou, no lado esquerdo, hipoplasia do osso ilíaco e do fêmur, ausência da tíbia com duplicação da fíbula, e presença de sete metatarsos e dedos, sem um hálux identificável, no pé. A ecocardiografia identificou um defeito do septo atrial. Acreditamos, com base na literatura, que o espectro de anormalidades apresentado por nossa paciente possa estar relacionado com o efeito disruptivo vascular do misoprostol. Entretanto, não podemos excluir a possibilidade de que essa associação possa ter sido simplesmente uma coincidência.

## INTRODUCTION

Mirror polydactyly is a rare type of hand or foot polydactyly that is characterized by mirror-image duplication around a midline axis on the arm or leg, with the absence of a recognizable thumb or hallux.^1^ Although extremely rare, mirror polydactyly of the foot may be associated with tibial agenesis and fibular dimelia.^2^ On the other hand, VACTERL (costovertebral segmentation defects, anal atresia/stenosis, cardiac malformation, tracheoesophageal fistula and/or esophageal atresia, renal and limb anomalies) (OMIM 192350)^*^ is an acronym for a nonrandom association of congenital anomalies for which the pathogenesis and etiology are still poorly understood.^3^

We report here on a girl with unilateral fibular dimelia and mirror polydactyly of the foot that presented together with features of the VACTERL association.

## CASE REPORT

The patient was a seven-month-old white girl who was the third child of young, healthy and non-consanguineous parents. The family history was unremarkable with regard to congenital defects. The patient was born at 40 weeks of gestational age, by vaginal delivery, with cephalic presentation, weighing 2,560 g (P3-10), measuring 47 cm (P3-10), with head circumference of 34.5 cm (P50-98) and Apgar scores of 2 and 7 at the first and fifth minutes, respectively. Her mother said that she had used misoprostol in the second month of pregnancy, at a single dose of 600 μg (three tablets) orally, with the objective of inducing abortion. She presented abdominal cramps and slight vaginal bleeding soon after taking misoprostol. She also drank alcohol in small amounts and smoked an average of three cigarettes a day throughout the pregnancy. Her glycemia was normal.

The child presented respiratory failure and needed tracheal intubation soon after birth. An imperforate anus was identified at the first evaluation, and colostomy was performed on the first day of life. In addition, genitography revealed an associated rectourethral fistula. Difficulty in nasogastric tube insertion was also noted. Esophageal atresia with a tracheoesophageal fistula was identified from an esophagogram. This was surgically corrected on the second day of life.

**Figure 1.**
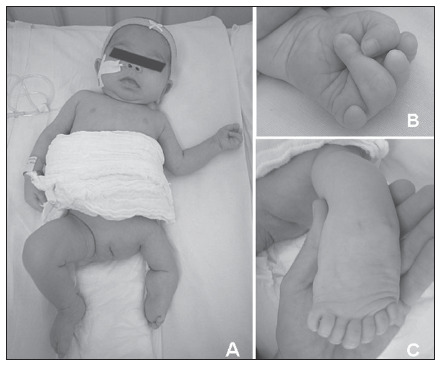
Patient at seven months of age showing anteverted nares, long philtrum and hemihypoplasia (A), a left-hand reduction defect with absence of the extremities of the second, third and fifth fingers (B) and mirror polydactyly of the foot (C).

At the age of seven months, her body weight was 3,320 g (P < 3), height 58 cm (P < 3) and head circumference 37.5 cm (P < 2). She had anteverted nares and a long philtrum (**Figure 1A**). No facial palsy or involvement of other cranial nerves was observed. Her left hand presented a reduction defect, with absence of the extremities of the second, third and fifth fingers and camptodactyly of the fourth finger (**Figure 1B**). The ipsilateral lower limb presented significant shortening, especially rhizomelic shortening. Her left foot presented a mirror configuration with seven toes and no identifiable hallux (**Figures 1A** **and C**). The right-side limbs were normal. The pelvis was hypoplastic, with hypoplasia of the labia majora. Neurological evaluation identified hypotonia.

**Figure 2.**
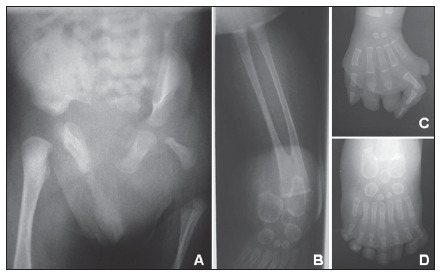
Radiographs showing hypoplasia of the left iliac bone and femur (A), left leg with absence of tibia and duplicated fibula (B), left hand with absence of distal phalanges of the second, third and fifth fingers (C) and mirror polydactyly of the foot (D).

A computed tomography scan of the head showed that the lateral ventricles were prominent. Abdominal ultrasound detected right kidney agenesis and left kidney pyelocaliceal duplication. Radiographic evaluation found agenesis of the distal and medial phalanges and hypoplasia of the proximal phalanges of the second and third fingers of the left hand. No phalanges were observed on the fifth finger. Scoliosis and partial sacral agenesis were observed in the spine. On the left side, iliac bone and femur were hypoplastic, the tibia was absent with a duplicated fibula and the foot presented duplicated tarsal bones and seven metatarsals and toes with no hallux (mirror polydactyly of the foot) (**Figure 2**). Echocardiography demonstrated an atrial septal defect. GTG-banded karyotyping (≥ 550 bands) gave normal results.

## DISCUSSION

Mirror foot is an uncommon anomaly that has often been described as an isolated defect. Its association with tibial agenesis and fibular dimelia is extremely rare^2,4,5^ (**Table 1**). This limb defect may be a feature of Laurin-Sandrow syndrome (OMIM 135750), a genetic disease that is characterized by mirror hands and striking facial features with a groove in the nasal columella. However, such findings were not observed in our patient.^6^ Some authors have suggested that there may be a correlation between mirror foot/fibular dimelia and teratogenic events. These would be expected to occur when the developmental specification reaches the level of the future knee, i.e. around the fifth week of development.^2^ Interestingly, this period also corresponds to the beginning of organogenesis and upper-limb differentiation, and this time correlated with the development of other abnormalities presented by our patient.^1^ In addition, it matched with the time at which the mother said she had used misoprostol.

**Table 1.** Results from our review of the medical databases using descriptors for the main clinical findings observed in our patient

Database	Search strategy	Results*
PubMed	"Fibular dimelia" AND "mirror foot polydactyly"	3 case reports (Bayram et al.^4^; Rivera et al.^2^; Verghese et al.^5^) No reviews of the literature
	"Fibular dimelia" AND "mirror foot polydactyly" AND "VACTERL", or "Fibular dimelia" AND "VACTERL", or "mirror foot polydactyly" AND "VACTERL"	No case reports or reviews of the literature

* Using the same search strategies in the Cochrane Library, SciELO and Lilacs databases, no results were found.

VACTERL has been defined as a multiple polytopic developmental field defect. Limb abnormalities are frequent under such conditions and they particularly involve radial rays. Tibial hypoplasia/aplasia with or without additional tibial field defects is the most common type of lower-limb malformation. Femoral abnormalities (such as hypoplasia or aplasia) and polydactyly also have been described and frequently produce asymmetric impairment, as observed in our patient.^3^ Although specific findings of mirror foot and fibular dimelia have never been described in VACTERL cases (**Table 1**), this is considered to be an abnormality secondary to a tibial field defect. Furthermore, asymmetric impairment to the developing embryo, as observed in our patient, is common in the VACTERL association.^3^

Misoprostol is a synthetic analogue of prostaglandin E1 that is widely misused in Brazil as an abortifacient. It is known that its vascular disruptive effect during the first trimester of pregnancy can lead to fetal malformations that especially affect craniofacial structures and limbs.^7^ However, its association with the pattern of abnormalities observed in our patient had never been described previously.

Some authors have suggested that such associations may result from teratogenic events that occur during organogenesis.^8^ Our patient presented esophageal atresia with a tracheoesophageal fistula, congenital heart defect, imperforate anus and unilateral renal agenesis, which are findings that characterize the VACTERL association. Interestingly, these features have also been associated with gestational exposure to vascular disruption agents, including maternal diabetes^9^ and even misoprostol.^10^ In a study conducted in Brazil, Nascimento et al.^10^ found a statistically significant correlation between such defects and gestational exposition to this drug. Our patient also presented asymmetric limb abnormalities that are frequently observed in cases of vascular disruptive lesions, such as exposure to misoprostol.^1,11^

These findings suggest that the spectrum of malformations presented by our patient may be related to the vascular disruptive effect of misoprostol during pregnancy. However, we cannot rule out the possibility that this association might simply be a coincidence.

## Footnotes

* Online Mendelian Inheritance in Man. Available from: http://www.ncbi.nlm.nih.gov/omim. Accessed in 2010 (Feb 11).
